# A Perspective on Using Virtual Reality to Incorporate the Affective Context of Everyday Falls Into Fall Prevention

**DOI:** 10.2196/36325

**Published:** 2023-01-11

**Authors:** Tiphanie E Raffegeau, William R Young, Peter C Fino, A Mark Williams

**Affiliations:** 1 School of Kinesiology George Mason University Manassas, VA United States; 2 School of Sport and Health Sciences University of Exeter Exeter United Kingdom; 3 Department of Health and Kinesiology University of Utah Salt Lake City, UT United States; 4 Health, Performance, and Resilience Group Institute of Human and Machine Cognition Pensacola, FL United States

**Keywords:** aging, balance, perturbation, locomotion, cognition, exergame, anxiety

## Abstract

Virtual reality (VR) is a promising and cost-effective tool that has the potential to reduce the prevalence of falls and locomotor impairments in older adults. However, we believe that existing VR-based approaches to prevent falls do not mimic the full breadth of perceptual, cognitive, and motor demands that older adults encounter in daily life. Researchers have not yet fully leveraged VR to address affective factors related to fall risk, and how stressors such as anxiety influence older adult balance and real-world falls. In this perspective paper, we propose developing VR-based tools that replicate the affective demands of real-world falls (eg, crossing the street) to enhance fall prevention diagnostics and interventions by capturing the underlying processes that influence everyday mobility. An effort to replicate realistic scenarios that precipitate falls in VR environments will inform evidence-based diagnostics and individualize interventions in a way that could reduce falls in older adults in daily life.

## Introduction

Annually, 1 in 4 older adults are injured from falling [1], and the incidence rates [2] and resulting morbidities continue to rise [3]. Alongside rapidly advancing virtual reality (VR) technology, scientists and clinicians are working to predict and prevent falls using a range of nonimmersive and immersive techniques [4-6]. Yet, researchers are only beginning to understand the potential benefits of VR technologies and their capabilities to target the perceptual, cognitive, and motor processes related to fall risk [7]. We believe there is a disconnect between how VR is currently used to understand and prevent falls in experimental settings and its capacity to identify and target the processes that are involved when older adults fall in daily life.

We argue that scientists currently using VR technology to evaluate and modify fall risk often overlook stressors such as anxiety or the “fear of falling” that are associated with higher prevalence of falls [8,9]. As we age, we use more attentional capacity while walking and stepping becomes less “automatic” [10]; thus, the added cognitive demand of fear of falling could be detrimental to balance and gait for older people [11-14]. Our underlying argument is that the context of balance control is important, and scientists should aim to create paradigms that better replicate the challenges that older adults experience during everyday walking to understand and prevent falls. By not representing the affective context that exists in the real world in VR-based tasks and interventions, we fail to address the interactions between perceptual, cognitive, and motor processes that underlie effective or maladaptive balance in daily life [15-18]. Scientists now have a unique opportunity to develop VR-based tools that facilitate interactions with realistic mobility contexts that induce anxiety as in the real world (eg, crossing a busy street at night and walking in a crowded mall). By using VR to engage with realistic contexts, we could better identify older adults who are most at risk of falling and develop refined interventions to prevent falls.

Our overarching interests are to establish early diagnostics that identify who is at greatest risk of falling and develop specific interventions that reduce the risk of falls in everyday life. In this perspective paper, we discuss leveraging the rising popularity of VR to develop meaningful protocols for diagnosing and treating the risk of falling in older adults. Specifically, we discuss how VR simulations can recreate the typical perceptual, cognitive, motor, and affective demands of daily life to facilitate diagnostics and interventions that reduce the risk of falling. The key points discussed in this paper are shown in Textbox 1.

**Key points discussed in this perspective.**

- Scientists should aim to better replicate the challenges that older adults experience during everyday walking to understand and prevent falls.
- We do not yet know if the experimental outcomes emerging from laboratory-based studies are representative of behavior in everyday life. However, virtual reality (VR) technology allows us to probe mobility-related affective responses with threats to stability and balance control.
- VR can simulate everyday situational demands and quantify responses with high resolution, thereby helping to bridge the gap between laboratory-based research outcomes and everyday fall risk.
- We propose a focus on scenarios where older individuals experience a fear of falling to identify associated deficits in perceptual, cognitive, and motor processes.
- To better detect everyday fall risk, scientists should incrementally challenge sensory, motor, and cognitive systems using a contextually appropriate VR-based stress test for fall risk.
- Using VR to address individual differences by measuring learning trajectories allows for tailored challenge points and appropriate difficulty levels that optimize learning.
- VR therapies successfully reduce anxiety in younger people; it is not hard to imagine a future where VR could help older adults overcome a fear of falling and cope with mobility-related anxiety.

## The Problem: Research on Fear of Falling and Fall Risk in Older Adults

As a result of poor “balance confidence” or low “falls efficacy,” many older adults report a “fear of falling” and experience mobility-related anxiety during locomotion [16]. We provide a brief overview of how fear and anxiety influence fall risk (ie, the physiological and cognitive response to a perceived threat in a balance or walking task). We refer the reader elsewhere for detailed reviews that distinguish these processes from concepts such as concern about falling [13,16,18]. Older adults who experience a fall are more likely to exhibit gait impairments and suffer higher incidents of falling [19,20]. We define “mobility-related anxiety” as a phobia specific to walking that is associated with increased physiological arousal and cognitive stress, which interferes with perceptual, cognitive, and motor processes during walking in young and older adults [12,21-24]. Both self-reported fear of falling and mobility-related anxiety are believed to impair cognitive-motor control, predisposing older adults to a greater risk of slips, trips, and falls [13,14,18]. However, there may be some protective element to mobility-related anxiety, encouraging older adults to compensate for poor balance by “consciously monitoring” their actions in efforts to remain safe [17]. The degree that mobility-related anxiety impedes or protects older adult balance is still unclear because current evidence is only supported by cross-sectional associations between fear of falling and motor outcomes [25], fall incidences [26], or anxiety-related responses from experimental manipulations (eg, raising participants on a platform) [11,23,27]. The experimental tasks used by scientists and clinicians are strictly controlled, and consequently, these tasks may not induce anxiety in a way that represents the anxiety-inducing experiences of older adults in real-world mobility scenarios (eg, crossing a busy street at night). We argue that scientists should leverage advances in technology to develop representative mobility tasks within VR simulations while simultaneously retaining experimental control. VR presents an ideal tool to create realistic contexts that would enhance and individualize fall-risk detection and prevention.

## Current Ways to Use VR to Study Mobility-Related Anxiety

Scientists interested in the effects of anxiety on mobility have predominantly used VR to create controlled experiments that answer fundamental science questions. Unfortunately, often the experimental conditions induced have limited relation to situations encountered in daily life. For example, our previous work has shown that immersive VR can be used to increase mobility-related anxiety in healthy people by simulating standing on a wooden plank and raising the walkway approximately 15 meters above ground level [24,28]. Although our approach allowed us to answer important questions about anxiety and motor control, we are hesitant to equate our results to behaviors in daily life. Historically, obtaining results that are representative of everyday tasks, or “representative task design,” has been a persistent limitation across many fields [29,30]. We do not yet know if the experimental outcomes emerging from controlled, laboratory-based studies are representative of behavior in everyday life. However, existing VR technology lends itself to probing affective responses by systematically imposing mobility-related threats to stability and testing balance control across external task constraints.

Scientists have typically used VR to induce mobility-related anxiety by replicating laboratory-based studies that physically raise participants to elevated heights [23,27,31]. The aim is to stimulate affective systems by increasing the perceived consequences of falling, also known as “postural threat” [23,31]. Simulating elevated heights in VR elicits similar changes to state anxiety (ie, subjective ratings) and motor behavior (ie, smaller postural sway area) as in the real world [32-34]. Various types of threatening contexts can elicit anxiety-related responses, ranging from simulated heights in a replicated laboratory environment [33,35,36] to riding an open elevator [37] and walking across a deep pit [38,39].

Another approach to study mobility-specific anxiety is to increase the difficulty of the locomotor task by walking on a narrow path. By increasing attentional demand and the energy cost of gait patterns [40,41], a narrower path imposes a demanding locomotor constraint that requires participants to continuously adapt and monitor their steps. A narrower step width also involves intrinsic risk appraisal by challenging stability; participants must evaluate their balance and devote added attentional resources to compensate for their anticipated missteps [42]. We interpret the added cognitive load of taking narrower steps as analogous to the “conscious monitoring” experienced by older adults who are anxious while walking [13,43].

VR-based tools can combine environmental and task-specific influences on mobility-related anxiety, emphasizing their potential to systematically examine the interactions between key perceptual, cognitive, and motor behaviors. Walking in VR on broad and narrow roads, and at low and high elevations, can reveal complex interactions between anxiety, cognition, and motor behavior in young and older adults [44]. For instance, combining path width and height manipulations in VR increases motor difficulty in addition to multiplying to consequences of a fall, forcing participants to prioritize performing the task safely versus quickly, revealing the interactions between mobility-related anxiety and motor performance [44]. Challenging balance in threatening VR environments (an increasingly narrowing path) reveals that balance confidence mediates the efficacy of older adults’ stepping responses [45,46]. Cumulatively, these results highlight the salience of mobility-related anxiety in locomotor behavior and its potential importance in improving balance and reducing fall risk.

However, while VR has led to advances in understanding mobility-related anxiety and balance, there remains a disconnect between experimental manipulations of anxiety responses and the lived experience of older adults suffering from mobility-related anxiety in daily life. We propose that researchers use VR to simultaneously test the influence of task- and environmentally driven affective responses on locomotion, while working to develop generalizable applications. We envision VR could be used as a tool to generate outcomes that are experimentally controlled, while simultaneously being representative of everyday contexts that precipitate real-world falls.

## Current Ways to Use VR-Based Technology for Fall Prevention

### Overview

While our perspective details ways to enhance the application of VR for fall prevention, existing empirical evidence already supports the use of VR-based tools for preventing falls in older adults. We overview the current use of VR technology in fall-prevention research with reference to the following three general categories of current technology: (1) nonimmersive VR; (2) augmented reality (AR); and (3) immersive VR. We consider the advantages and disadvantages of each approach with regard to older adult fall prevention.

### Nonimmersive VR

The majority of published reports focusing on the benefits of VR-based tools in older adult fall prevention use nonimmersive devices that range in design and application [47-54]. Nonimmersive technology typically delivers a gamified rehabilitation goal and provides 2D visual feedback of body position or motor performance on a television display. Position detection capabilities vary and include simpler platform or controller-based devices, such as a Wii balance board or Wii Fit (Nintendo), as well as optical tracking systems ranging from the X-box Kinect (Microsoft) to high-speed infrared cameras using expensive motion capture techniques such as the CAREN (Motek). Visual stimuli range from a low-cost flat-screen television presented at eye level [55] to digital projections onto a treadmill belt [56], to a 180 curved floor-to-ceiling projection screen [45,57,58]. Interactions within VR afford many options for providing biofeedback [59], where the majority of nonimmersive video games present both feedback of body position and knowledge of performance outcomes [6,55]. However, a lack of consistency in delivery and application of visual biofeedback in immersive VR [24,38,60] makes it difficult to determine if nonimmersive therapeutic protocols and positive effects are replicable or might be enhanced with advanced technology [61,62]. One significant issue relating to standardization of protocols relates to limited accessibility, as many nonimmersive VR tools are no longer commercially available. Nonimmersive VR applications therefore require custom programming, rendering the product commercially unscalable and only suitable for research purposes.

### Augmented Reality

AR, or mixed reality, is a unique technology with great potential to train balance and reduce falls using ecologically valid locomotor tasks. AR overlays 3D virtual illusions through interactive games within the individual’s real-world setting, typically using face-worn eyeglasses or goggles. Integrating the game into the real-world setting and feedback of oneself likely enhances the “task specificity” of AR-based tools for transferring performance to real-world balance and gait tasks [63]. AR has also shown promise in providing social interaction and psychological support for older people; its primary advantage for health-related applications may be its capacity to increase motivation to engage in physical training [63,64]. Perhaps a result of shifting gaze to look at or look through a peripheral display, lateral stability is compromised by AR glasses [65], which may be particularly detrimental for older adult fallers who are worse at controlling mediolateral balance [66]. AR technology shows great promise for everyday fall prevention, but development is lagging with respect to user-friendly programming and commercial accessibility, especially for older adults [63].

### Immersive VR

Typically delivered via a head-worn virtual display, or head-mounted display (HMD), immersive VR replaces one’s current setting with an interactive 2D (eg, video-based) or 3D (eg, digitally rendered) environment. By blocking out sensory feedback from the outside world, immersive VR presents an opportunity to stimulate sensory, and motor systems for fall prevention. Advances in graphic design and rendering capabilities have drastically improved the realism of immersive VR simulations and multiplied the potential experiences representative of daily life. Impaired older adults (ie, immobile or cognitively impaired) can experience 2D images using an HMD (usually video-based stimuli), without negative side effects such as motion sickness [67]. An HMD obstructs visual feedback of body position without added accessories or specific game development, which influences the control of basic perception and action [68] and locomotor control in VR simulations [62]. We speculate that ongoing bodily feedback may be a key difference between immersive and projection-based treadmill VR environments, facilitating a greater sense of immersion (measured as self-reported “presence”) during an elevated height simulation [69,70], and higher levels of reported anxiety in projection-based approaches when compared to an immersive HMD [71,72]. Table 1 presents the advantages and disadvantages of different VR types to prevent falls.

**Table 1:** Advantages and disadvantages of different types of virtual reality (VR) to prevent falls.

VR types	Advantages	Disadvantages
Nonimmersive VR	Feasible for use with older adults in community settings [51] and people with Parkinson disease [73] Benefits impaired populations such as stroke survivors [74,75] and people with Parkinson disease [76,77] Interventions improve performance on physical function tests related to fall risk [52,53,78], reduce fear of falling and depression [79,80], and reduce the incidence of falls in frail older adults [78] Can replicate balance perturbation training techniques with visual perturbations [35,58] that improve balance recovery and reduce real-world falls [81,82].	A lack of consistency in delivery or application of visual biofeedback (ie, displaying motor performance versus results, or both) make it difficult to determine if therapeutic results are replicable Most nonimmersive VR equipment is no longer commercially available and requires custom programming that is only suitable for research purposes
AR^a^	AR interactions take place in everyday settings and can serve as realistic substitutes for environmental manipulations or cues. Placing AR obstacles in one’s path simulates the task of avoiding them as if they existed in reality, but without the trip hazard [83] AR-based visual and audio cues often improve gait characteristics in people with neurological conditions, particularly people with Parkinson disease [84]	Lateral stability is compromised by AR glasses [65], which may be particularly detrimental for older adults prone to falling or those with deficits in controlling mediolateral balance [66] Older adults report current AR goggles as heavy and uncomfortable during balance training [85], an issue that could be addressed by forthcoming technology mimicking traditional spectacles Development is lagging with respect to user-friendly programming and commercial accessibility, especially for older adults [63]
Immersive VR	Can systematically implement visual perturbations (ie, shifting the room suddenly) that induce a sensation of falling [86], leading to a decline in stability and forcing older adults to practice and train reactive balance recovery in a safer way [87,88] Using immersive VR to manipulate visual input during clinical physical function tests better detects fall risk [89]	Immersive HMD^b^ technology can be difficult to operate [90] Bulky and uncomfortable to wear [91] Limiting the visual field of view [92] Sometimes causing motion sickness [93] that could be interpreted as a sign of a potential fall by at-risk older adults HMDs obstruct visual feedback of body position without added hardware or customized programming to track body position. The absence of visual feedback influences basic perception and action [68], locomotor control [62], and interactions between anxiety and motor control in anxiety-inducing VR simulations [94]

^a^AR: augmented reality.

^b^HMD: head-mounted display.

## Incorporating the Context of Everyday Falls Into Fall Prevention

Rehabilitation scientists are limited in their ability to replicate complex everyday scenarios and elicit representative anxiety, hindering the ecological validity and translation of existing approaches. Simultaneously, we have yet to find a way to measure perceptual, cognitive, and motor processes in the real world without using disruptive and resource-heavy equipment. To address both limitations, VR can simulate everyday situational demands and quantify responses with high resolution, thereby helping to bridge the gap between laboratory-based research outcomes and everyday fall risk. As opposed to evaluating physical function in sterile clinical settings with clear hallways, using VR to challenge older adult mobility within an ecologically valid setting would better reveal real-world mobility deficits, thereby potentially leading to enhanced therapies to reduce fall risk.

Crossing the street at night is an example of a locomotor context that elicits a specific mobility-related anxiety and requires effective perceptual (eg, judging the distance to oncoming hazards with low-quality visual feedback), cognitive (eg, gathering and retaining limited visual and spatial information in working memory to monitor position), and motor adaptations (ie, neuromuscular coordination and step targeting) to achieve safely. It may therefore be no surprise that older adults experience a higher prevalence of traffic-related injuries compared to younger pedestrians; a consequence attributed to a host of individual, task, and environmental factors [95], including self-reported fear of falling [96]. The capability of VR-based tools to replicate a street-crossing simulation for older adults was demonstrated in a series of studies using a projection-based room with a 360° simulation of a busy street and crosswalk [97], allowing people to move through a realistic 3D visual surround. The results show when older pedestrians avoid moving traffic, their cognitive-motor “workload” is higher than when crossing an empty street, leading older adults to walk faster and ignore traffic-avoidance–related tasks [98]. These latter behaviors are reminiscent of older adults in artificial anxiety-inducing settings (ie, a narrow or elevated walkway) [44]. A similar study recently used a street-crossing paradigm in an immersive commercial HMD apparatus measuring eye tracking, locomotor behavior, and cognitive performance to draw inferences about pedestrian behaviors in young adults [99]. Such studies highlight the potential to use VR to simulate realistic everyday contexts that challenge affective responses while being feasible for older adult users. We believe further efforts are needed to develop similar immersive, yet effective, VR-based approaches using simulations of real-world contexts where falls are common.

The greater challenge is to capture behavior from fearful older adults during threatening walking scenarios and best use VR to quantify behaviors that perpetuate falls. We propose a focus on scenarios where older individuals experience a fear of falling, especially in settings leading to real-world falls such as stairwells, escalators, crowded sidewalks, shopping malls, or nighttime streets. Borrowing from the concepts developed from successful trauma-focused VR-based treatment of posttraumatic stress disorder (PTSD) in soldiers [99,100], combat scenarios are predictable sources of phobic anxiety. Therefore, presenting a realistic combat scenario in VR is likely to elicit a phobic response. Yet, we cannot be certain which scenarios cause anxiety responses for older adults in real life and how those vary individually. We propose qualitative interview-based inquiry [101], a type of “needs assessment” or “task analysis,” as a step toward determining the scenarios that are anxiety inducing for older adults with motor impairments related to a concern about falling. Our previous qualitative work interviewed fearful older adults to examine their thoughts during hypothetical scenarios leading to falls, and our findings debunked popular theories about attentional focus (ie, attentional control theory) under anxiety or stress developed using data from athletes [102]. Whereas athletes are distracted by a "threat bias" in attentional control that is maladaptive in competitive settings [103], we showed that some degree of "conscious monitoring" dring walking is protective for older adults who realistically appraise their sense of control in the situation [17]. Only individuals who expressed lacking a sense of control demonstrated a maladaptive fear of falling [17], highlighting the value of qualitative inquiry in translating theory into population-specific interventions.

VR environments that represent everyday situations associated with a fear of falling would allow clinicians to examine locomotor performance on a range of functional tasks in a fall risk "stress test". Such a virtual “stress test” was recently demonstrated in a functional VR game using an urban scenario designed for rehabilitating activities of daily living [104]. The “UrbanRehab” VR tool was created to optimize ecological validity and challenge outdoor mobility in urban settings to train and enhance movement. The creators of the UrbanRehab program began with a focus group of rehabilitation specialists and aimed to create an environment where patients could be challenged with a variety of different tasks in a realistic cityscape. The challenges and achievements of the UrbanRehab program are reported from a developer’s perspective in further detail [104], highlighting the development-related challenges of designing and implementing realistic VR games for rehabilitation. For example, designing realistic games that enable functional movement is limited by in-home space constraints, presenting a common and difficult challenge that developers must overcome. However, the UrbanRehab initiative represents an exciting first step in realizing the potential for VR to reduce real-world falls.

A major advantage of VR is the capability to address individual differences due to its capacity to change the level of task difficulty and create appropriate “challenge points” or “desirable difficulties” [105]. To better detect everyday fall risk, a range of typical sensory, motor, and cognitive challenge points can be incorporated into a contextually appropriate VR-based stress test for fall risk. For instance, incorporating increasingly physiologically demanding locomotor tasks as challenge points, such as changes in walking speed along a crosswalk, could better reveal fall risk related to poor lower limb strength or power [106] and may be a better predictor of fall risk than measuring walking at comfortable speeds [107]. VR-based challenge points can present increasingly difficult but realistic obstacles for avoidance such as potholes, puddles, or oncoming pedestrians [108], which would better identify people who struggle to maintain balance during complex stepping tasks [109]. Challenging balance control in VR with dynamic locomotor tasks such as turning would reveal deficits in motor control mechanisms that are relevant to everyday falls [110,111]. Using challenge points to progressively manipulate visual flow during walking [46,57] or walking in increasingly low-light settings [112] would flag sensory integration deficits for intervention (ie, proper corrective eye wear, visuo-motor training) before they become a problem [113]. Finally, presenting increasingly complicated but realistic concurrent cognitive tasks as challenge points, such as concurrently monitoring a clock or oncoming pedestrians [99], would better identify older people at risk of real-world falls due to cognitive-motoric deficits [114,115]. In sum, VR allows scientists to measure performance across different challenge points, whereby participants are pushed to perform at, or near, the limits of their capability in everyday cognitive-motor performance, highlighting locomotor deficits before they lead to a fall in daily life.

The individual differences in learning outcomes detected across challenge points in diagnostics can optimize VR-based interventions for each learner and lead to enhanced transfer of skills to the real world. VR lends itself to quantifying and adjusting training loads for individual responses to an intervention and augmenting training loads for learners [105]. Commercially available VR systems boast multiple ways to measure performance (eg, eye tracking and kinematics) to achieve goal-related outcomes (eg, a gamified performance goal), and either aspect of a simulation can be tailored to target individual needs and training goals. Advances in data modeling and machine learning allow us to measure individual differences in learning by analyzing changes in performance versus goal-related outcomes (ie, speed versus accuracy analyses) across repeated practice sessions or challenge points. Using VR to quantify individual learning trajectories allows for tailored challenge points and maintaining appropriate difficulty levels [105], and VR-based approaches could adjust training loads to optimize learning for each participant throughout an intervention.

Practitioners are already using VR with younger populations to rehabilitate those with anxiety-related disorders, pioneering a wider application of VR for “exposure therapy” to reduce anxiety [100]. For example, the Bravemind program builds on evidence highlighting that VR-based exposure therapy can reduce depression and anxiety in veteran soldiers with PTSD [116]. Bravemind uses VR to simulate combat-related scenarios in combination with cognitive behavioral therapy, which reframes anxiety-based reactions with “cognitive restructuring,” providing a low-threat context where a patient can therapeutically decondition the cycle of anxiety via habituation [100]. The immersive VR program trains soldiers to compensate and adapt to their PTSD symptoms over time with prolonged exposure to threatening stimuli in VR, guided by evidence from successful VR treatments leveraging exposure therapy for specific phobias [69,117]. Similar exposure effects are reported in VR-based anxiety-inducing settings; the effects of mobility-related anxiety on motor performance (ie, gait speed) decay as participants acclimate to the simulated scenario [23,24], suggesting a similar approach may work to train older adults at risk for maladaptive mobility-related anxiety.

The use of VR-based approaches in conjunction with cognitive behavioral therapy could be effective in reducing fear of falling in older adults [118]. VR-based therapies for treating anxiety disorders are rising in popularity, and meta-analyses support using VR for treating anxiety when compared with traditional therapies [119], especially in preventing patient attrition [119,120]. While no substantive advantages are associated with current VR-based programs compared to traditional therapy, participants are more likely to adhere to VR-based therapies, thus improving their efficacy. We speculate that higher levels of adherence could be a result of greater engagement, which improves motor learning outcomes in gamified rehabilitation programs as compared to the sterility of traditional rehabilitation settings [64]. If VR technology can be refined and harnessed, we believe that the effectiveness (and relative affordability) of such therapies can outperform traditional treatment methods. Because of the success of programs such as Bravemind and the increasingly immersive VR experiences, it is not difficult to imagine a future where older adults are trained to optimize their performance on everyday mobility tasks using controlled exposure to stressful virtual environments.

## Conclusions

VR is an appealing and practical tool to enhance and maintain older adult mobility. We envision a future where realistic contexts for individualized VR-based fall-prevention diagnostics and interventions will reduce the risk of real-world falls in older adults. By incorporating the context of daily tasks into VR-based approaches, we can quantify movement within complex and realistic environments that better represent the demands that older adults navigate every day. Focusing on using VR to mimic the influence of affect on cognitive, perceptual, and motor behaviors should guide industry, researchers, and clinicians toward therapies that prevent everyday falls.

## Abbreviations

AR: augmented reality
HMD: head-mounted display
PTSD: posttraumatic stress disorder
VR: virtual reality

## References

[1] (2019). STEADI - Older Adult Fall Prevention. Centers for Disease Control and Prevention.

[2] Bergen G, Stevens MR, Burns ER (2016). Falls and Fall Injuries Among Adults Aged ≥65 Years - United States, 2014. MMWR Morb Mortal Wkly Rep.

[3] Burns E, Kakara R (2018). Deaths from Falls Among Persons Aged ≥65 Years - United States, 2007-2016. MMWR Morb Mortal Wkly Rep.

[4] Pacheco TBF, de Medeiros CSP, de Oliveira VHB, Vieira ER, de Cavalcanti FAC (2020). Effectiveness of exergames for improving mobility and balance in older adults: a systematic review and meta-analysis. Syst Rev.

[5] Mirelman A, Deutsche J, Hausdorff J (2014). Virtual reality augmented training for improving walking and reducing fall risk in patients with neurodegenerative disease. Virtual Real. Phys. Mot. Rehabil.

[6] Mirelman A, Rochester L, Maidan I, Del Din S, Alcock L, Nieuwhof F, Rikkert MO, Bloem BR, Pelosin E, Avanzino L, Abbruzzese G, Dockx K, Bekkers E, Giladi N, Nieuwboer A, Hausdorff JM (2016). Addition of a non-immersive virtual reality component to treadmill training to reduce fall risk in older adults (V-TIME): a randomised controlled trial. Lancet.

[7] Corregidor-Sánchez AI, Segura-Fragoso A, Rodríguez-Hernández M, Jiménez-Rojas C, Polonio-López B, Criado-Álvarez JJ (2021). Effectiveness of virtual reality technology on functional mobility of older adults: systematic review and meta-analysis. Age Ageing.

[8] Gazibara T, Kurtagic I, Kisic-Tepavcevic D, Nurkovic S, Kovacevic N, Gazibara T, Pekmezovic T (2017). Falls, risk factors and fear of falling among persons older than 65 years of age. Psychogeriatrics.

[9] Friedman SM, Munoz B, West SK, Rubin GS, Fried LP (2002). Falls and fear of falling: which comes first? A longitudinal prediction model suggests strategies for primary and secondary prevention. J Am Geriatr Soc.

[10] Hoang I, Paire-Ficout L, Derollepot R, Perrey S, Devos H, Ranchet M (2022). Increased prefrontal activity during usual walking in aging. Int J Psychophysiol.

[11] Ellmers TJ, Cocks AJ, Young WR (2020). Exploring attentional focus of older adult fallers during heightened postural threat. Psychol Res.

[12] Holtzer R, Kraut R, Izzetoglu M, Ye K (2019). The effect of fear of falling on prefrontal cortex activation and efficiency during walking in older adults. Geroscience.

[13] Young WR, Mark Williams A (2015). How fear of falling can increase fall-risk in older adults: applying psychological theory to practical observations. Gait Posture.

[14] Hadjistavropoulos T, Carleton RN, Delbaere K, Barden J, Zwakhalen S, Fitzgerald B, Ghandehari OO, Hadjistavropoulos H (2012). The relationship of fear of falling and balance confidence with balance and dual tasking performance. Psychol Aging.

[15] Young WR, Ellmers TJ, Kinrade NP, Cossar J, Cocks AJ (2020). Re-evaluating the measurement and influence of conscious movement processing on gait performance in older adults: Development of the Gait-Specific Attentional Profile. Gait Posture.

[16] Ellmers TJ, Wilson MR, Kal EC, Young WR (2022). Standing up to threats: Translating the two-system model of fear to balance control in older adults. Exp Gerontol.

[17] Ellmers TJ, Wilson MR, Norris M, Young WR (2022). Protective or harmful? A qualitative exploration of older people's perceptions of worries about falling. Age Ageing.

[18] Hadjistavropoulos T, Delbaere K, Fitzgerald TD (2011). Reconceptualizing the role of fear of falling and balance confidence in fall risk. J Aging Health.

[19] Eckert T, Kampe K, Kohler M, Albrecht D, Büchele G, Hauer K, Schäufele M, Becker C, Pfeiffer K (2020). Correlates of fear of falling and falls efficacy in geriatric patients recovering from hip/pelvic fracture. Clin Rehabil.

[20] Young WR, Olonilua M, Masters RSW, Dimitriadis S, Mark Williams A (2016). Examining links between anxiety, reinvestment and walking when talking by older adults during adaptive gait. Exp Brain Res.

[21] Gage WH, Sleik RJ, Polych MA, McKenzie NC, Brown LA (2003). The allocation of attention during locomotion is altered by anxiety. Exp Brain Res.

[22] Brown LA, Polych MA, Doan JB (2006). The effect of anxiety on the regulation of upright standing among younger and older adults. Gait Posture.

[23] Adkin AL, Carpenter MG (2018). New insights on emotional contributions to human postural control. Front Neurol.

[24] Raffegeau TE, Fawver B, Clark M, Engel BT, Young WR, Williams AM, Lohse KR, Fino PC (2020). The feasibility of using virtual reality to induce mobility-related anxiety during turning. Gait Posture.

[25] Young WR, Wing AM, Hollands MA (2012). Influences of state anxiety on gaze behavior and stepping accuracy in older adults during adaptive locomotion. J Gerontol B Psychol Sci Soc Sci.

[26] Landers MR, Oscar S, Sasaoka J, Vaughn K (2016). Balance Confidence and Fear of Falling Avoidance Behavior Are Most Predictive of Falling in Older Adults: Prospective Analysis. Phys Ther.

[27] Brown LA, Gage WH, Polych MA, Sleik RJ, Winder TR (2002). Central set influences on gait. Age-dependent effects of postural threat. Exp Brain Res.

[28] Raffegeau TE, Fawver B, Young WR, Williams AM, Lohse KR, Fino PC (2020). The direction of postural threat alters balance control when standing at virtual elevation. Exp Brain Res.

[29] Williams AM, Ericsson KA (2005). Perceptual-cognitive expertise in sport: some considerations when applying the expert performance approach. Hum Mov Sci.

[30] Araújo D, Davids K, Passos P (2007). Ecological Validity, Representative Design, and Correspondence Between Experimental Task Constraints and Behavioral Setting: Comment on Rogers, Kadar, and Costall (2005). Ecological Psychology.

[31] Adkin AL, Frank JS, Carpenter MG, Peysar GW (2000). Postural control is scaled to level of postural threat. Gait Posture.

[32] Bohil CJ, Alicea B, Biocca FA (2011). Virtual reality in neuroscience research and therapy. Nat Rev Neurosci.

[33] Cleworth TW, Horslen BC, Carpenter MG (2012). Influence of real and virtual heights on standing balance. Gait Posture.

[34] Meehan M, Insko B, Whitton M, Brooks FP (2002). Physiological measures of presence in stressful virtual environments. ACM Trans. Graph.

[35] Cleworth TW, Chua R, Inglis JT, Carpenter MG (2016). Influence of virtual height exposure on postural reactions to support surface translations. Gait Posture.

[36] Boroomand-Tehrani A, Huntley AH, Jagroop D, Campos JL, Patterson KK, Tremblay L, Mansfield A (2020). The effects of postural threat induced by a virtual environment on performance of a walking balance task. Hum Mov Sci.

[37] Martens MA, Antley A, Freeman D, Slater M, Harrison PJ, Tunbridge EM (2019). It feels real: physiological responses to a stressful virtual reality environment and its impact on working memory. J Psychopharmacol.

[38] Peterson S, Furuichi E, Ferris D (2018). Effects of virtual reality high heights exposure during beam-walking on physiological stress and cognitive loading. PLoS One.

[39] Ehgoetz Martens KA, Ellard CG, Almeida QJ (2015). Virtually-induced threat in Parkinson's: Dopaminergic interactions between anxiety and sensory-perceptual processing while walking. Neuropsychologia.

[40] Donelan JM, Shipman DW, Kram R, Kuo AD (2004). Mechanical and metabolic requirements for active lateral stabilization in human walking. J Biomech.

[41] Clark DJ (2015). Automaticity of walking: functional significance, mechanisms, measurement and rehabilitation strategies. Front Hum Neurosci.

[42] Kal EC, Young WR, Ellmers TJ (2022). Balance capacity influences the effects of conscious movement processing on postural control in older adults. Hum Mov Sci.

[43] Ellmers TJ, Young WR (2018). Conscious motor control impairs attentional processing efficiency during precision stepping. Gait Posture.

[44] Schaefer S, Schellenbach M, Lindenberger U, Woollacott M (2015). Walking in high-risk settings: do older adults still prioritize gait when distracted by a cognitive task?. Exp Brain Res.

[45] Kazanski ME, Dingwell JB (2021). Effects of age, physical and self-perceived balance abilities on lateral stepping adjustments during competing lateral balance tasks. Gait Posture.

[46] Kazanski ME, Cusumano JP, Dingwell JB (2020). How healthy older adults regulate lateral foot placement while walking in laterally destabilizing environments. J Biomech.

[47] Neri SG, Cardoso JR, Cruz L, Lima RM, de Oliveira RJ, Iversen MD, Carregaro RL (2017). Do virtual reality games improve mobility skills and balance measurements in community-dwelling older adults? Systematic review and meta-analysis. Clin Rehabil.

[48] Li J, Erdt M, Chen L, Cao Y, Lee S, Theng Y (2018). The Social Effects of Exergames on Older Adults: Systematic Review and Metric Analysis. J Med Internet Res.

[49] Molina KI, Ricci NA, de Moraes SA, Perracini MR (2014). Virtual reality using games for improving physical functioning in older adults: a systematic review. J Neuroeng Rehabil.

[50] Marston HR, Smith ST (2012). Interactive Videogame Technologies to Support Independence in the Elderly: A Narrative Review. Games Health J.

[51] Miller KJ, Adair BS, Pearce AJ, Said CM, Ozanne E, Morris MM (2014). Effectiveness and feasibility of virtual reality and gaming system use at home by older adults for enabling physical activity to improve health-related domains: a systematic review. Age Ageing.

[52] Rendon AA, Lohman EB, Thorpe D, Johnson EG, Medina E, Bradley B (2012). The effect of virtual reality gaming on dynamic balance in older adults. Age Ageing.

[53] Kamińska MS, Miller A, Rotter I, Szylińska A, Grochans E (2018). The effectiveness of virtual reality training in reducing the risk of falls among elderly people. Clin Interv Aging.

[54] Smith ST, Schoene D (2012). The use of exercise-based videogames for training and rehabilitation of physical function in older adults: current practice and guidelines for future research. Aging Health.

[55] Mirelman A, Rochester L, Reelick M, Nieuwhof F, Pelosin E, Abbruzzese G, Dockx K, Nieuwboer A, Hausdorff JM (2013). V-TIME: a treadmill training program augmented by virtual reality to decrease fall risk in older adults: study design of a randomized controlled trial. BMC Neurol.

[56] Potocanac Z, Smulders E, Pijnappels M, Verschueren S, Duysens J (2015). Response inhibition and avoidance of virtual obstacles during gait in healthy young and older adults. Hum Mov Sci.

[57] Franz JR, Francis CA, Allen MS, O'Connor SM, Thelen DG (2015). Advanced age brings a greater reliance on visual feedback to maintain balance during walking. Hum Mov Sci.

[58] Mccrum C Research report: Gait Perturbation Research using the CAREN. Motek.

[59] Levin MF, Sviestrup H, Subramanian SK (2010). Feedback and virtual environments for motor learning and rehabilitation. Schedae.

[60] Janeh O, Bruder G, Steinicke F, Gulberti A, Poetter-Nerger M (2018). Analyses of Gait Parameters of Younger and Older Adults During (Non-)Isometric Virtual Walking. IEEE Trans Vis Comput Graph.

[61] Bauer A, Andringa G (2020). The Potential of Immersive Virtual Reality for Cognitive Training in Elderly. Gerontology.

[62] Kim A, Kretch KS, Zhou Z, Finley JM (2018). The quality of visual information about the lower extremities influences visuomotor coordination during virtual obstacle negotiation. J Neurophysiol.

[63] Lee LN, Kim MJ, Hwang WJ (2019). Potential of Augmented Reality and Virtual Reality Technologies to Promote Wellbeing in Older Adults. Applied Sciences.

[64] Lohse KR, Boyd LA, Hodges NJ (2016). Engaging Environments Enhance Motor Skill Learning in a Computer Gaming Task. J Mot Behav.

[65] Sedighi A, Rashedi E, Nussbaum MA (2020). A head-worn display ("smart glasses") has adverse impacts on the dynamics of lateral position control during gait. Gait Posture.

[66] Nordin E, Moe-Nilssen R, Ramnemark A, Lundin-Olsson L (2010). Changes in step-width during dual-task walking predicts falls. Gait Posture.

[67] Appel L, Appel E, Bogler O, Wiseman M, Cohen L, Ein N, Abrams HB, Campos JL (2019). Older Adults With Cognitive and/or Physical Impairments Can Benefit From Immersive Virtual Reality Experiences: A Feasibility Study. Front Med (Lausanne).

[68] Day B, Ebrahimi E, Hartman LS, Pagano CC, Robb AC, Babu SV (2019). Examining the effects of altered avatars on perception-action in virtual reality. J Exp Psychol Appl.

[69] Krijn M, Emmelkamp PM, Biemond R, de Wilde de Ligny C, Schuemie MJ, van der Mast CA (2004). Treatment of acrophobia in virtual reality: The role of immersion and presence. Behaviour Research and Therapy.

[70] Gromer D, Reinke M, Christner I, Pauli P (2019). Causal Interactive Links Between Presence and Fear in Virtual Reality Height Exposure. Front Psychol.

[71] Juan MC, Pérez D (2009). Comparison of the Levels of Presence and Anxiety in an Acrophobic Environment Viewed via HMD or CAVE. Presence: Teleoperators and Virtual Environments.

[72] Gromer D, Madeira O, Gast P, Nehfischer M, Jost M, Müller M, Mühlberger A, Pauli P (2018). Height Simulation in a Virtual Reality CAVE System: Validity of Fear Responses and Effects of an Immersion Manipulation. Front Hum Neurosci.

[73] Perrochon A, Borel B, Istrate D, Compagnat M, Daviet J (2019). Exercise-based games interventions at home in individuals with a neurological disease: A systematic review and meta-analysis. Ann Phys Rehabil Med.

[74] Valdés BA, Glegg SMN, Lambert-Shirzad N, Schneider AN, Marr J, Bernard R, Lohse K, Hoens AM, Van der Loos HFM (2018). Application of Commercial Games for Home-Based Rehabilitation for People with Hemiparesis: Challenges and Lessons Learned. Games Health J.

[75] Lohse KR, Hilderman CGE, Cheung KL, Tatla S, Van der Loos HFM (2014). Virtual reality therapy for adults post-stroke: a systematic review and meta-analysis exploring virtual environments and commercial games in therapy. PLoS One.

[76] Canning CG, Allen NE, Nackaerts E, Paul SS, Nieuwboer A, Gilat M (2020). Virtual reality in research and rehabilitation of gait and balance in Parkinson disease. Nat Rev Neurol.

[77] Sultana M, Bryant D, Orange JB, Beedie T, Montero-Odasso M (2020). Effect of Wii Fit© Exercise on Balance of Older Adults with Neurocognitive Disorders: A Meta-Analysis. J Alzheimers Dis.

[78] Fu AS, Gao KL, Tung AK, Tsang WW, Kwan MM (2015). Effectiveness of Exergaming Training in Reducing Risk and Incidence of Falls in Frail Older Adults With a History of Falls. Arch Phys Med Rehabil.

[79] Yang J, Lee T, Kim J (2017). The effect of a VR exercise program on falls and depression in the elderly with mild depression in the local community. J Phys Ther Sci.

[80] Zeng N, Pope Z, Lee JE, Gao Z (2018). Virtual Reality Exercise for Anxiety and Depression: A Preliminary Review of Current Research in an Emerging Field. J Clin Med.

[81] McCrum C, Gerards MHG, Karamanidis K, Zijlstra W, Meijer K (2017). A systematic review of gait perturbation paradigms for improving reactive stepping responses and falls risk among healthy older adults. Eur Rev Aging Phys Act.

[82] McCrum C, Bhatt T, Gerards M, Karamanidis K, Rogers M, Lord S (2022). Perturbation-based Balance Training: Principles, Mechanisms and Implementation in Clinical Practice. OSF Preprints.

[83] Coolen B, Beek PJ, Geerse DJ, Roerdink M (2020). Avoiding 3D Obstacles in Mixed Reality: Does It Differ from Negotiating Real Obstacles?. Sensors (Basel).

[84] Lachica I, Chan T, Ph D, Vicente J, Ayala M, Krause A (2020). A novel augmented reality platform delivers sensory cues to improve Parkinsonian gait.

[85] Blomqvist S, Seipel S, Engström M (2021). Using augmented reality technology for balance training in the older adults: a feasibility pilot study. BMC Geriatr.

[86] Anson E, Critelli K, Chen E, Staab J, Carpenter M, Crane B (2022). Did I sway? Yes, No, Maybe so?. International Society for Gait and Posture Conference Proceedings.

[87] Delgado F, Der Ananian C (2021). The Use of Virtual Reality Through Head-Mounted Display on Balance and Gait in Older Adults: A Scoping Review. Games Health J.

[88] Parijat P, Lockhart TE, Liu J (2015). Effects of perturbation-based slip training using a virtual reality environment on slip-induced falls. Ann Biomed Eng.

[89] Almajid R, Tucker C, Wright WG, Vasudevan E, Keshner E (2020). Visual dependence affects the motor behavior of older adults during the Timed Up and Go (TUG) test. Arch Gerontol Geriatr.

[90] Kourtesis P, Collina S, Doumas LAA, MacPherson SE (2019). Technological Competence Is a Pre-condition for Effective Implementation of Virtual Reality Head Mounted Displays in Human Neuroscience: A Technological Review and Meta-Analysis. Front Hum Neurosci.

[91] Park S, Lee G (2020). Full-immersion virtual reality: Adverse effects related to static balance. Neurosci Lett.

[92] Nielsen E (2020). Characterizing visually evoked postural responses with a virtual reality head-mounted display in young and older adults. PhD Thesis. University of British Columbia.

[93] Akiduki H, Nishiike S, Watanabe H, Matsuoka K, Kubo T, Takeda N (2003). Visual-vestibular conflict induced by virtual reality in humans. Neurosci Lett.

[94] Quek DYL, Economou K, MacDougall H, Lewis SJG, Ehgoetz Martens KA (2022). The influence of visual feedback on alleviating freezing of gait in Parkinson's disease is reduced by anxiety. Gait Posture.

[95] Wilmut K, Purcell C (2022). Why Are Older Adults More at Risk as Pedestrians? A Systematic Review. Hum Factors.

[96] Avineri E, Shinar D, Susilo YO (2012). Pedestrians' behaviour in cross walks: the effects of fear of falling and age. Accid Anal Prev.

[97] Cavallo V, Dommes A, Dang N, Vienne F (2019). A street-crossing simulator for studying and training pedestrians. Transportation Research Part F: Traffic Psychology and Behaviour.

[98] Dommes A (2019). Street-crossing workload in young and older pedestrians. Accid Anal Prev.

[99] Rizzo A, Hartholt A, Grimani M, Leeds A, Liewer M (2014). Virtual Reality Exposure Therapy for Combat-Related Posttraumatic Stress Disorder. Computer.

[100] Rizzo A, Shilling R (2017). Clinical Virtual Reality tools to advance the prevention, assessment, and treatment of PTSD. Eur J Psychotraumatol.

[101] Timsina LR, Willetts JL, Brennan MJ, Marucci-Wellman H, Lombardi DA, Courtney TK, Verma SK (2017). Circumstances of fall-related injuries by age and gender among community-dwelling adults in the United States. PLoS One.

[102] Eysenck MW, Derakshan N (2011). New perspectives in attentional control theory. Personality and Individual Differences.

[103] Wilson M (2008). From processing efficiency to attentional control: a mechanistic account of the anxiety–performance relationship. International Review of Sport and Exercise Psychology.

[104] Juan-González J, García AS, Molina JP, López-Jaquero V, Navarro E, Romero-Ayuso D, González P (2021). UrbanRehab: a virtual urban scenario design tool for rehabilitating instrumental activities of daily living. J Ambient Intell Human Comput.

[105] Anderson DI, Lohse KR, Lopes TCV, Williams AM (2021). Individual differences in motor skill learning: Past, present and future. Hum Mov Sci.

[106] Baudendistel ST, Schmitt AC, Stone AE, Raffegeau TE, Roper JA, Hass CJ (2021). Faster or longer steps: Maintaining fast walking in older adults at risk for mobility disability. Gait Posture.

[107] Middleton A, Fulk GD, Herter TM, Beets MW, Donley J, Fritz SL (2016). Self-Selected and Maximal Walking Speeds Provide Greater Insight Into Fall Status Than Walking Speed Reserve Among Community-Dwelling Older Adults. Am J Phys Med Rehabil.

[108] Liu W, Zhang J, Li X, Song W (2022). Avoidance behaviors of pedestrians in a virtual-reality-based experiment. Physica A: Statistical Mechanics and its Applications.

[109] Geerse DJ, Roerdink M, Marinus J, van Hilten JJ (2019). Walking adaptability for targeted fall-risk assessments. Gait Posture.

[110] Mancini M, Schlueter H, El-Gohary M, Mattek N, Duncan C, Kaye J, Horak FB (2016). Continuous Monitoring of Turning Mobility and Its Association to Falls and Cognitive Function: A Pilot Study. J Gerontol A Biol Sci Med Sci.

[111] Crenshaw JR, Bernhardt KA, Achenbach SJ, Atkinson EJ, Khosla S, Kaufman KR, Amin S (2017). The circumstances, orientations, and impact locations of falls in community-dwelling older women. Arch Gerontol Geriatr.

[112] Rand KM, Creem-Regehr SH, Thompson WB (2015). Spatial learning while navigating with severely degraded viewing: The role of attention and mobility monitoring. J Exp Psychol Hum Percept Perform.

[113] Lord SR (2006). Visual risk factors for falls in older people. Age Ageing.

[114] Li KZH, Bherer L, Mirelman A, Maidan I, Hausdorff JM (2018). Cognitive Involvement in Balance, Gait and Dual-Tasking in Aging: A Focused Review From a Neuroscience of Aging Perspective. Front Neurol.

[115] Allali G, Launay CP, Blumen HM, Callisaya ML, De Cock A-M, Kressig RW, Srikanth V, Steinmetz J, Verghese J, Beauchet O, Biomathics Consortium (2017). Falls, Cognitive Impairment, and Gait Performance: Results From the GOOD Initiative. J Am Med Dir Assoc.

[116] Beidel DC, Frueh BC, Neer SM, Bowers CA, Trachik B, Uhde TW, Grubaugh A (2019). Trauma management therapy with virtual-reality augmented exposure therapy for combat-related PTSD: A randomized controlled trial. J Anxiety Disord.

[117] Rothbaum BO, Hodges L, Smith S, Lee JH, Price L (2000). A controlled study of virtual reality exposure therapy for the fear of flying. J Consult Clin Psychol.

[118] Liu T, Ng GYF, Chung RCK, Ng SSM (2018). Cognitive behavioural therapy for fear of falling and balance among older people: a systematic review and meta-analysis. Age Ageing.

[119] Fodor LA, Coteț CD, Cuijpers P, Szamoskozi S, David D, Cristea IA (2018). The effectiveness of virtual reality based interventions for symptoms of anxiety and depression: A meta-analysis. Sci Rep.

[120] Benbow AA, Anderson PL (2019). A meta-analytic examination of attrition in virtual reality exposure therapy for anxiety disorders. J Anxiety Disord.

